# New-onset constipation at acute stage after stroke: incidence, risk factors, and impact on stroke rehabilitation

**DOI:** 10.3389/fneur.2026.1721157

**Published:** 2026-04-15

**Authors:** Zhaoyang Lv, Shiyu Fan, Xv Ma, Feng Liu, Hao Wu, Zhihong Shi, Meng Wang, Tao Yu, Pan Wang, Shuai Liu, Yong Ji

**Affiliations:** 1 Department of Neurology, Tianjin Key Laboratory of Cerebral Blood Flow Reconstruction and Head and Neck Tumor New Technology Translation, Tianjin Key Laboratory of Cerebral Vascular and Neurodegenerative Diseases, Huanhu Hospital Affiliated to Tianjin Medical University, Tianjin, China; 2 National Clinical Medical Research Center of Acupuncture, The First Affiliated Hospital of Tianjin University of Traditional Chinese Medicine, Tianjin, China

**Keywords:** constipation, cross-sectional study, rehabilitation, risk factors, stroke

## Abstract

**Background:**

The rapid increase in the global aging population poses a major public health challenge due to the prevalence of stroke. Constipation represents a significant complication of stroke, significantly impacting quality of life. The objective of this study is to investigate the incidence of constipation in acute stroke and to identify the factors that contribute to new-onset constipation (defined as constipation developing after stroke in patients with no prior history) after stroke.

**Methods:**

A total of 600 acute stroke patients were recruited for a cross-sectional study, with a questionnaire administered to each participant. This study included demographic characteristics, stroke type and focus, medical history, sleep quality, psychological problems, and the NIHSS and Barthel index (BI). Participants with constipation were evaluated for severity, medications, and stroke outcomes at discharge by the investigators.

**Results:**

Of the 600 acute stroke patients, 126 (21%) reported a history of constipation. Furthermore, 278 patients (46.3%) demonstrated post-stroke constipation (PSC), while 184 patients (38.8%) experienced new-onset constipation following their stroke. The results indicated that hemorrhagic stroke, posterior circulation stroke, diabetes, osmotic diuretics, antacids, use of bedpans, Difficulty falling asleep, depression, and a higher NIHSS score at admission were significant risk factors for new-onset constipation. In patients demonstrating moderate severity, PSC correlated with adverse stroke outcomes at discharge.

**Conclusion:**

The incidence of constipation in acute stroke patients is higher than that in the general population. The results suggest that depression and Difficulty falling asleep may increase the risk of new-onset constipation. Moreover, after adjusting for confounders, new-onset constipation was independently associated with poor discharge outcome, particularly in patients with moderate stroke severity. Early identification of constipation risk in stroke patients can improve the development and optimization of rehabilitation protocols.

**Clinical trial registration:**

Identifier ChiCTR2400080663.

## 1 Introduction

Stroke is classified as a condition characterized by rapidly progressive focal or global neurological deficits, excluding vascular origins and other identifiable causes, leading to clinical symptoms that persist for over 24 h or result in death. Stroke shows a significant incidence, leading to higher rates of disability, recurrence, and mortality (1). According to the Global Burden of Disease Study, in 2019, approximately 12.2 million new stroke cases were reported globally (95% UI, 11–13.6 million), leading to a total of 101 million individuals affected by stroke worldwide (95% UI, 93.2–111 million). Stroke is the second leading cause of death worldwide, representing 11.6% of total fatalities (2). The findings underscore the necessity for further studies on early stroke prevention and immediate post-stroke intervention. Further, certain post-stroke complications, including gastrointestinal diseases, frequently influence stroke prognosis. The results of a previous study indicate a possible association between constipation and stroke (3).

Constipation is a prevalent gastrointestinal disorder, affecting 10.1 to 15.3% of neurologically healthy adults (4). The prevalence of post-stroke constipation (PSC) among stroke patients ranges from 22.9 to 60% (5), influenced by variables such as age, gender, and the diagnostic criteria applied. Furthermore, individuals suffering from constipation are 19% more likely to experience an ischemic stroke compared to those without constipation (6), and PSC significantly affects the prognosis of those with stroke. Patients with PSC may engage in excessive effort during defecation, resulting in elevated abdominal and intracranial pressure. This may lead to a second stroke or cause defecation syncope, myocardial infarction, or brain herniation, all of which pose significant risks to the patient’s life and recovery process (7). Moreover, long-term retention of intestinal contents can lead to substantial absorption of intestinal toxins, which can, in turn, exacerbate cerebral circulation disorders such as stroke.

Therefore, constipation frequently results in diminished quality of life for patients and prolonged hospitalization after a stroke, placing a significant strain on both the patient and their family or caregiver (8). However, the mechanisms underlying PSC are not yet fully understood. Being bedridden and dehydrated, reduced consciousness, and side effects of medication may all contribute to the development of PSC (9). Furthermore, prolonged colonic transit time decreased colonic contractions and reduced gastrointestinal motility in stroke patients, perhaps contributing to PSC (10). In this study, we define PSC as constipation occurring after stroke, regardless of pre-existing history. New-onset constipation refers specifically to a subset of PSC occurring in patients with no prior history of constipation. This distinction is maintained throughout the manuscript to ensure clarity in risk factor analysis and outcome assessment. Accordingly, the present study aims to investigate the incidence of constipation in patients with acute stroke, identify risk factors for new-onset constipation, and examine the association between new-onset constipation and functional outcome at discharge, with the goal of informing preventive strategies and rehabilitation care.

## 2 Materials and methods

This study was approved by the Ethics Committee of Huanhu Hospital in Tianjin, and all procedures involving participants were conducted following the institution’s guidelines. All participants provided both oral and written informed consent. This study was conducted following the Helsinki Declaration and received approval from the Ethical Review Board of Tianjin Huanhu Hospital [(Jinhuan) Luncheon Review Nos. (2023-157) and (2024-175)]. Moreover, the present study was registered on the Chinese Clinical Trial Registry website, bearing registration number ChiCTR2400080663.^1^

### 2.1 Inclusion and exclusion criteria

This study investigated the eligibility of patients with acute stroke admitted to the hospital from January 1, 2024, to July 1, 2024. Inclusion criteria: (1) Age ≥ 18 years, and (2) hospitalization within 7 days post-stroke onset. A stroke is determined based on the criteria established by the World Health Organization and validated using CT or MRI imaging. Exclusion criteria: (1) History of neurological or psychiatric disorders, (2) death within 7 days after stroke onset, (3) history of severe liver or kidney disease or malignant tumors, (4) history of rectal or colonic structural diseases, and (5) consciousness disorders or other impairments affecting the investigation.

### 2.2 Diagnosis of constipation

The diagnostic criteria for constipation were based on the Rome III criteria for International Functional Gastrointestinal Disorders (11): At least two of the following symptoms should be present: (1) Straining during defecation, (2) lumpy or hard stools, (3) a feeling of incomplete evacuation, (4) sensation of anorectal obstruction or blockage, (5) manual maneuvers to facilitate defecation (e.g., digital disimpaction, pelvic floor support, etc.), (6) defecation frequency less than three times per week, the occurrence of criteria 1 through 5 at least 25% of the time, (7) infrequent loose stools without the use of laxatives, (8) not meeting the diagnostic criteria for Irritable Bowel Syndrome (IBS).

### 2.3 Predictors of PSC

A survey was performed to establish predictors of constipation following a stroke. The survey employed questionnaires that encompassed the following: fundamental demographic attributes such as gender, age, educational attainment, marital status, living conditions, smoking and alcohol consumption history, dietary practices, stroke type, stroke focus, mobility status, medical history (including hypertension, diabetes, coronary heart disease, etc.), medication usage, and defecation posture. Furthermore, the severity of the condition was evaluated in cases where participants demonstrated symptoms of constipation. The duration of patients’ nighttime sleep was examined along with sleep disorders, anxiety, and depressive symptoms. The NIHSS score and Barthel index (BI) were used to evaluate the severity of the patient’s functional impairment post-stroke. The research team completed the questionnaires, which were then assessed and examined by trained neurologists, with support from gastroenterologists. Every staff member who evaluated the patient’s functional impairment underwent the same training at Tianjin Huanhu Hospital.

### 2.4 Assessment of stroke outcome

Vascular death and handicap were indicative of a poor stroke outcome. Deaths occurring during hospitalization that were associated with stroke, myocardial infarction, other cardiac diseases, or sudden death were classified as vascular deaths (12).

Handicap was evaluated using the BI (13). This scale quantifies the degree of disability by assessing the patient’s ability to complete daily activities and live independently. This metric is well-established in stroke research and is understood as a comprehensive measure of functional health, emphasizing physical impairment. Participants were classified into two groups according to their BI score: dependent (BI score ≤60) and independent (BI score >60) (14). Patients with a dependency score were identified as being disabled at discharge.

### 2.5 Statistical analysis

Data were analyzed using SPSS 27.0. Continuous variables were tested for normality; normally distributed data are presented as mean ± SD and compared by independent t-tests, while non-normally distributed data are expressed as median (interquartile range) and analyzed with Mann–Whitney U tests. Categorical variables were compared using χ^2^ or Fisher’s exact tests. To identify independent risk factors for new-onset constipation, variables with *p* < 0.05 in univariate analysis were entered into multivariable binary logistic regression. Continuous predictors (age, NIHSS) were assessed for linearity in the logit using the Box–Tidwell test (no violation), and multicollinearity was evaluated with variance inflation factors (all VIF < 2). The model was constructed using forward stepwise selection, while clinically important covariates (stroke type, stroke focus, diabetes, osmotic diuretics, antacids, bedpan use, Difficulty falling asleep, depression, NIHSS) were forced into the final model based on prior literature; gender and residency were adjusted *a priori* due to their known associations with constipation and stroke outcomes (15, 16). To evaluate whether new-onset constipation independently predicted poor discharge outcome (Barthel Index ≤ 60 or vascular death), multivariable logistic regression was performed with new-onset constipation as the primary independent variable. Covariates included age, sex, stroke type, history of constipation, admission NIHSS, and admission Barthel Index. Three sequential models were constructed: Model 1 (unadjusted); Model 2 (adjusted for stroke severity: admission NIHSS and Barthel Index); and Model 3 (fully adjusted: Model 2 plus age, sex, stroke type, and history of constipation). Subgroup analyses were performed stratifying patients by baseline NIHSS (mild: 0–3; moderate: 4–11; severe: 12–29). Adjusted odds ratios (OR) and 95% confidence intervals (CI) were calculated, with two-sided *p* < 0.05 considered statistically significant. Missing data were minimal (<5%) and handled by complete-case analysis.

## 3 Results

**Figure 1:**
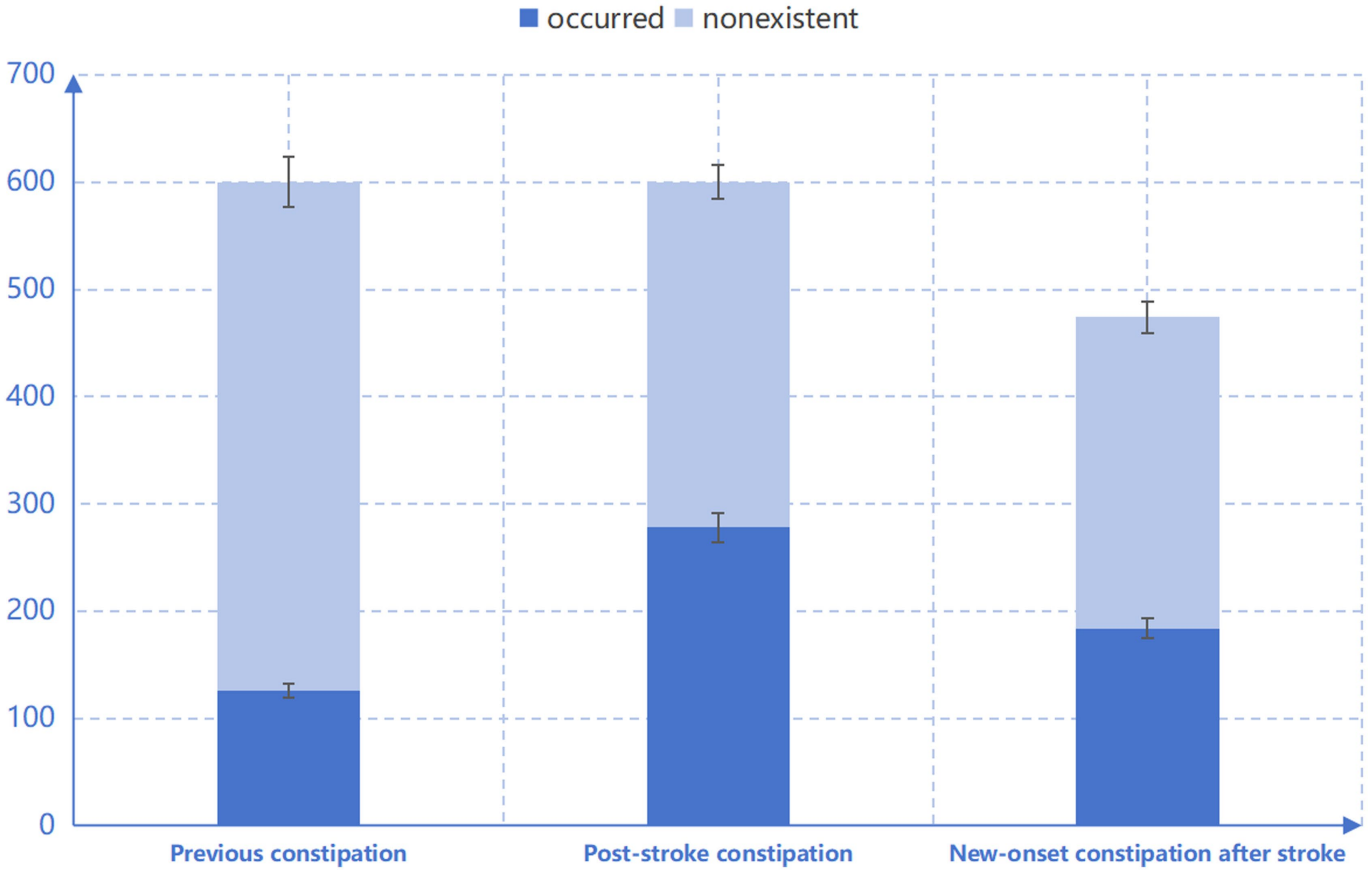
Occurrence of constipation in acute stroke patients.

Six hundred stroke patients participated in the survey. The average age was 63 years, and the median length of hospitalization was 6 days. A total of 424 stroke patients (70.6%) were male, and 126 patients (21%) had a history of constipation. A total of 278 patients, representing 46.3%, experienced PSC. The incidence of new-onset constipation was 184 (38.8%) (Figure 1).

### 3.1 The effect of pre-existing constipation at acute stroke

The findings of this study revealed a slightly higher incidence rate of past constipation (21%) than that of chronic constipation in the older population in general. For this group, constipation lasted, on average, 4 years. Patients with stroke were separated into two groups according to their constipation history: those who had never constipated and those who had experience. Differences in demographic characteristics were observed between the two groups. Males, those who are married, those who are living with a spouse, and those with an education level above junior high school were less likely to have a history of constipation. Elderly individuals, females, widowed persons, and those cohabiting with children showed a higher susceptibility to constipation. Individuals with a history of constipation displayed a higher likelihood of experiencing Difficulty falling asleep. There was a negative correlation with the post-stroke BI score and a positive correlation with the post-stroke NIHSS score.

### 3.2 Incidence of PSC

Among 600 patients with acute stroke, 278 experienced constipation post-stroke, representing 46.3%. The mean onset time for constipation was 2 days following hospital admission. Patients were categorized into two groups according to the presence of constipation post-admission: those without constipation following the stroke and those with constipation following the stroke. This study identified differences among the groups regarding demographic characteristics, stroke type, stroke focus, medical history, medication use, mobility, sleep quality, emotional issues, and BI and NIHSS scores upon admission. Patients with primary school education and below, widowhood, hemorrhage stroke, posterior circulation stroke, diabetes as comorbidity, use of osmotic diuretics, anticonvulsants, antidepressants, and antacids, those who were bedridden, those who defecated in a potty, Difficulty falling asleep, daytime sleepiness, depression, and lower BI and higher NIHSS score at admission were more likely to develop PSC (Table 1).

**Table 1:** Demographic and clinical information collection in PSC.

Variant	Non-constipation after stroke (*n* = 322)	PSC (*n* = 278)	*p*-value
Age (mean ± sd)	60.82 ± 0.644	66.06 ± 0.653	<0.001***
Educational level (year)	9 (3,15)	9 (6,12)	0.021*
Primary and below (*n*, %)	88 (27.3)	102 (36.7)	0.014*
Junior high school (*n*, %)	141 (43.8)	107 (38.5)	0.189
Senior high school (*n*, %)	81 (25.1)	60 (21.6)	0.303
University and above (*n*, %)	12 (3.7)	9 (3.2)	0.745
Duration of hospitalization (days) [Median (Q1, Q3)]	6 (4,8)	6 (4,8)	0.07*
Gender, Male (*n*, %)	231 (71.7)	193 (69.4)	0.535
Smoking (*n*, %)	175 (54.3)	142 (51.1)	0.424
Alcohol consumption (*n*, %)	131 (40.7)	112 (40.3)	0.922
Stroke type			
Ischemic stroke (*n*, %)	298 (92.5)	225 (80.9)	<0.001***
Hemorrhagic stroke (*n*, %)	16 (5.0)	50 (18.0)	<0.001***
Stroke focus			
Anterior circulation (*n*, %)	157 (50.9)	106 (38.7)	0.012*
Posterior circulation (*n*, %)	119 (37.9)	135 (49.3)	0.004**
Medications			
Osmotic diuretics (*n*, %)	26 (8.1)	67 (24.1)	<0.001***
Antacids (*n*, %)	42 (13)	83 (29.9)	<0.001***
Anticonvulsants (*n*, %)	14 (4.3)	26 (9.4)	0.014*
Antidepressants (*n*, %)	4 (1.2)	15 (5.4)	0.004**
Antihistamines (*n*, %)	8 (2.5)	11 (4.0)	0.304
Past medical history			
Hypertension (*n*, %)	235 (73)	211 (75.9)	0.415
Diabetes (*n*, %)	102 (31.7)	120 (43.2)	<0.001***
Coronary heart disease (*n*, %)	53 (16.5)	51 (18.3)	0.543
Arrhythmia (*n*, %)	14 (4.3)	10 (3.6)	0.640
Marital status			
Spouse (*n*, %)	279 (86.6)	229 (82.4)	0.148
Widowhood (*n*, %)	33 (10.2)	45 (16.2)	0.031*
Bowel position			
Bedridden (*n*, %)	41 (12.7)	115 (41.4)	<0.001***
Bedside (*n*, %)	8 (2.5)	11 (4.0)	0.304
Toilet (*n*, %)	270 (83.9)	151 (54.3)	<0.001***
Diet			
Nasogastric medication administration (*n*, %)	11 (3.4)	35 (12.6)	<0.001***
Semiliquid diet (*n*, %)	5 (1.6)	8 (2.9)	0.266
General feeding (*n*, %)	306 (95.0)	232 (83.5)	<0.001***
Sleep			
Difficulty falling asleep (*n*, %)	38 (11.8)	72 (25.9)	<0.001***
Daytime sleepiness (*n*, %)	24 (7.5)	43 (15.5)	0.002**
Dysphylaxia (*n*, %)	47 (14.6)	51 (18.3)	0.215
RBD (*n*, %)	4 (1.2)	2 (0.7)	0.521
Anxiety (*n*, %)	146 (45.3)	142 (51.1)	0.161
Depression (*n*, %)	49 (15.2)	113 (40.8)	<0.001***
NIHSS score at admission (*n*, %)	2 (−1,5)	5 (−1,11)	<0.001***
Barthel Index at admission (*n*, %)	80 (50,110)	65 (15,115)	<0.001***

*p < 0.05, **p < 0.01, ***p < 0.001,the difference is statistically significant, NIHSS indicates National Institutes of Health Stroke Scale; *n*, No. of patients.

### 3.3 Incidence of new-onset constipation

Of the 474 patients with acute stroke and no prior history of constipation, 184 were diagnosed with new-onset constipation, representing 38.8% of the cohort. The mean duration for the onset of constipation was 2 days post-hospital admission. Only 5% of patients had a history of constipation, which improved following the stroke. Patients without a prior history of constipation were categorized into two groups: those experiencing new-onset constipation post-stroke and those who did not. Patients were classified into two groups according to the incidence of constipation following admission (Table 2). No significant differences were observed between the groups regarding gender, education level, marital status, and smoking or alcohol consumption. However, a higher percentage of patients in the new-onset constipation group were bedridden, received nasogastric tube feeding, and used bedpans. Patients with a history of diabetes, cerebral hemorrhage stroke, posterior circulation stroke, and those using osmotic diuretics, antidepressants, anticonvulsants, and antacids were more likely to develop new-onset constipation. Patients who engaged in daytime napping showed Difficulty falling asleep and depressive symptoms, and they faced an increased risk of developing new-onset constipation. A statistically significant difference (*p* < 0.05) was observed in the NIHSS score and BI at admission between individuals with and without new-onset constipation.

**Table 2:** Demographic and clinical information collection in new-onset constipation.

Variant	Non-new onset Constipation after stroke (*n* = 290)	New-onset constipation (*n* = 184)	*p*-value
Age (mean ± sd)	60.63 ± 0.677	64.38 ± 0.807	<0.001***
Educational level (Year)	9 (3,15)	9 (4,14)	0.129
Primary and below (*n*, %)	76 (26.2)	60 (32.6)	0.13
Junior high school (*n*, %)	128 (44.1)	78 (42.4)	0.71
Senior high school (*n*, %)	74 (25.5)	39 (21.2)	0.28
University and above (*n*, %)	12 (3.7)	9 (3.2)	0.86
Duration of hospitalization (days) [Median (Q1, Q3)]	6 (4,8)	6 (4,8)	0.073
Gender, Male (*n*, %)	214 (73.8)	133 (72.3)	0.72
Smoking (*n*, %)	157 (54.1)	97 (52.7)	0.76
Alcohol Consumption (*n*, %)	123 (42.4)	78 (42.4)	0.99
Stroke type			
Ischemic stroke (*n*, %)	268 (92.4)	142 (77.2)	<0.001***
Hemorrhagic stroke (*n*, %)	15 (5.2)	41 (22.3)	<0.001***
Stroke focus			
Anterior circulation (*n*, %)	144 (50.9)	73 (40.1)	0.034*
Posterior circulation (*n*, %)	107 (37.8)	88 (48.4)	0.018*
Medications			
Osmotic diuretics (*n*, %)	22 (7.6)	54 (29.3)	<0.001***
Antacids (*n*, %)	35 (12.1)	56 (30.4)	<0.001***
Anticonvulsants (*n*, %)	12 (4.1)	19 (10.3)	0.008**
Antidepressants (*n*, %)	2 (0.7)	6 (3.3)	0.034*
Antihistamines (*n*, %)	8 (2.8)	7 (3.8)	0.562
Past medical history			
Hypertension (*n*, %)	211 (72.8)	139 (75.5)	0.501
Diabetes (*n*, %)	87 (30.0)	81 (44.0)	0.002**
Coronary heart disease (*n*, %)	46 (15.9)	29 (15.8)	0.977
Arrhythmia (*n*, %)	14 (4.8)	7 (3.8)	0.598
Marital status			
Spouse (*n*, %)	256 (88.3)	157 (85.3)	0.350
Widowhood (*n*, %)	34 (11.7)	27 (14.7)	0.134
Bowel position			
Bedridden (*n*, %)	35 (12.1)	80 (43.5)	<0.001***
Bedside (*n*, %)	7 (2.4)	1 (0.5)	0.123
Toilet (*n*, %)	245 (84.5)	102 (55.4)	<0.001***
Diet			
Nasogastric medication administration (*n*, %)	8 (2.8)	22 (12.0)	<0.001***
Semiliquid diet (*n*, %)	5 (1.7)	7 (3.8)	0.16
General feeding (*n*, %)	277 (95.5)	155 (84.2)	<0.001***
Sleep			
Difficulty falling asleep (*n*, %)	31 (10.7)	43 (23.4)	<0.001***
Daytime sleepiness (*n*, %)	20 (6.9)	29 (15.8)	0.002**
Dysphylaxia (*n*, %)	42 (14.5)	35 (19)	0.192
RBD (*n*, %)	3 (1.0)	2 (1.1)	0.957
Anxiety (*n*, %)	127 (43.8)	92 (50)	0.187
Depression (*n*, %)	42 (14.5)	80 (43.7)	<0.001***
NIHSS score at admission (*n*, %)	2 (−1,5)	4 (−2,10)	<0.001***
Barthel Index at admission (*n*, %)	80 (50,110)	70 (20,120)	<0.001***

*p < 0.05, **p < 0.01, ***p < 0.001,the difference is statistically significant.

### 3.4 Predictors of new-onset constipation

A χ2 test was performed to determine categorical variables that best predicted new-onset constipation. The results indicated that elder age (*p* < 0.001), hemorrhagic stroke (*p* < 0.001), posterior circulation stroke (*p* = 0.018), bedridden status (*p* < 0.001), a history of diabetes (*p* = 0.002), use of anticonvulsant drugs (*p* = 0.008), osmotic diuretics (*p* < 0.001), antacids (*p* < 0.001), antidepressants (*p* = 0.034), Nasogastric medication administration (*p* < 0.001), and the use of bedpans (*p* < 0.001) were closely associated with the development of new-onset constipation. These results indicate that patients displaying daytime sleepiness (*p* = 0.002), Difficulty falling asleep (*p* < 0.001), depression (*p* < 0.001), a higher NIHSS score at admission (*p* < 0.001), and a lower BI at admission (*p* < 0.001) are at an increased risk of developing constipation post-stroke. Through a multi-factor binary logistic regression analysis, and after controlling for gender and residency, age, hemorrhagic stroke, posterior circulation stroke, diabetes, the use of osmotic diuretics and antacids, the use of bedpans, Difficulty falling asleep, depressive symptoms, and the NIHSS score at admission were identified as significant indicators of new-onset constipation (Table 3).

**Table 3:** Multi-factor binary logistic regression analysis on the association between new-onset constipation and significant factors.

Variables	OR	95% CI		*p*-value
		Lower limit	Higher limit	
Age (years)	1.025	1.002	1.048	0.029*
Cerebral hemorrhage	2.707	1.102	6.648	0.03*
Posterior circulation	1.729	1.076	2.78	0.024*
Diabetes	1.738	1.081	2.792	0.022*
Osmotic diuretics	2.746	1.235	6.102	0.013*
Antacids	1.937	1.054	3.562	0.033*
Use of bedpans	4.37	1.342	14.232	0.014*
Difficulty falling asleep	1.885	1.012	3.511	0.046*
Depression	4.863	2.892	8.176	<0.001***
NIHSS	1.143	1.04	1.256	0.006**

*p < 0.05, **p < 0.01, ***p < 0.001, the difference is statistically significant.

### 3.5 Association between new-onset constipation and poor discharge outcome

To investigate whether new-onset constipation independently predicts poor functional outcome at discharge, we performed multivariable logistic regression analyses adjusting for potential confounders. In the unadjusted model (Model 1), new-onset constipation was significantly associated with poor outcome (OR = 2.57, 95% CI: 1.77–3.74, *p* < 0.001). After adjusting for stroke severity (admission NIHSS and Barthel Index) in Model 2, the association was attenuated and became non-significant (OR = 2.61, 95% CI: 0.91–7.49, *p* = 0.074). However, after further adjustment for age, sex, stroke type, and history of constipation in the fully adjusted model (Model 3), new-onset constipation remained an independent predictor of poor discharge outcome (OR = 3.68, 95% CI: 1.54–8.84, *p* = 0.003). Admission Barthel Index (OR = 0.85, 95% CI: 0.82–0.88, *p* < 0.001) and history of constipation (OR = 3.14, 95% CI: 1.24–7.98, *p* = 0.016) were also independently associated with poor outcome in the final model (Table 4).

**Table 4:** Multivariable logistic regression analysis for poor outcome at discharge (all patients).

Variable	Model 1 (unadjusted)	Model 2 (adjusted for stroke severity)	Model 3 (fully adjusted)
New-onset constipation (yes vs. no)	2.57 (1.77–3.74) *p* < 0.001***	2.61 (0.91–7.49) *p* = 0.074	3.68 (1.54–8.84) *p* = 0.003**
Admission Barthel Index		0.85 (0.80–0.91) *p* < 0.001***	0.85 (0.82–0.88) *p* < 0.001***
Admission NIHSS score		0.62 (0.30–1.31) *p* = 0.209	0.97 (0.84–1.11) *p* = 0.639
Age (years)			1.02 (0.98–1.05) *p* = 0.370
Sex (female vs. male)			0.84 (0.37–1.89) *p* = 0.676
Stroke type (hemorrhagic vs. ischemic)			1.56 (0.63–3.90) *p* = 0.336
History of constipation (yes vs. no)			3.14 (1.24–7.98) *p* = 0.016*

Data are presented as odds ratio (95% confidence interval). Model 1 included only new-onset constipation. Model 2 adjusted for admission NIHSS and Barthel Index. Model 3 further adjusted for age, sex, stroke type, and history of constipation. *p < 0.05, **p < 0.01, ***p < 0.001.

**Figure 2:**
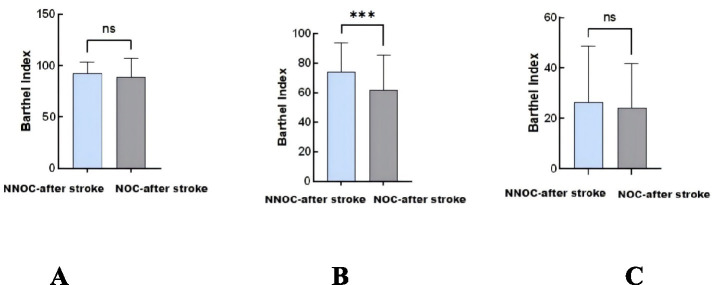
**(A–C)** Unadjusted comparison of discharge Barthel index between patients with and without new-onset constipation, stratified by baseline NIHSS severity. *p < 0.05, **p < 0.01, ***p < 0.001.

We further examined the association in subgroups stratified by baseline stroke severity (Table 5). Among patients with mild stroke (NIHSS 0–3), new-onset constipation was independently associated with poor outcome after full adjustment (OR = 2.50, 95% CI: 1.18–5.33, *p* = 0.017). The association was even stronger in the moderate stroke group (NIHSS 4–11; OR = 5.89, 95% CI: 1.58–21.99, *p* = 0.008). In the severe stroke group (NIHSS 12–29), no significant association was detected, likely due to the small sample size and consequent limited statistical power (OR = 0.20, 95% CI: 0.00–>999, *p* = 0.998) (Tables 4, 5; Figure 2).

**Table 5:** Subgroup analysis: association between new-onset constipation and poor outcome stratified by baseline NIHSS severity.

Stroke severity (NIHSS)	Adjusted OR (95% CI)	*p*-value
Mild (0–3)	2.50 (1.18–5.33)	0.017*
Moderate (4–11)	5.89 (1.58–21.99)	0.008**
Severe (12–29)	0.20 (0.00–>999)†	0.998

OR, odds ratio; CI, confidence interval. Adjusted for age, sex, stroke type, history of constipation, admission NIHSS, and admission Barthel Index. ^†^The extremely wide confidence interval in the severe group is due to the small sample size, resulting in unstable estimates. *p < 0.05, **p < 0.01, ***p < 0.001.

## 4 Discussion

This cross-sectional study of patients with acute stroke revealed a significantly higher incidence of PSC relative to the general population. Advanced age, bed rest, stroke type and focus, comorbid diabetes, use of osmotic diuretics and antacids, Difficulty falling asleep, depressive state, and elevated NIHSS score were significant predictors influencing the occurrence of PSC. Furthermore, the results indicate that PSC may negatively affect stroke outcomes.

### 4.1 Age and lifestyle habits

In the general population, constipation is more common in women and the elderly (17). The study revealed no correlation between gender and constipation within the acute stroke population. These findings indicate that advanced age correlates with an increased prevalence of constipation following a stroke, consistent with the data from previous research. Constipation risk increases with age due to diminished dietary intake and physical activity, reduced gastrointestinal secretion of digestive juices, impaired intestinal tone, and peristalsis, weakened anal sphincters, and unusual psychological behaviors (18).

The results indicate a strong correlation between constipation and patient mobility. Our multi-factor binary logistic regression analysis revealed that patients utilizing bedpans showed an increased likelihood of PSC. Inactivity leads to diminished intestinal motility, atrophied abdominal muscles, and a decline in muscle strength. This leads to extended fecal retention in the intestine, increased water absorption, and the formation of hard stools, which can readily induce or worsen constipation (19). Moreover, a lack of privacy in the defecation environment and the need for assistance in defecation can also negatively affect bowel movements.

### 4.2 Protective factors against PSC

Patients who did not develop constipation after stroke were characterized by better functional status, preserved mobility, and intact swallowing function. These observations suggest that early mobilization, maintaining independence in daily activities, and avoiding unnecessary medications may protect against PSC. Additionally, the lower prevalence of depression and sleep disturbances in the non-constipated group highlights the potential role of psychological well-being, consistent with emerging evidence linking the brain-gut-microbiota axis to bowel function (20). Physical activity has been shown to reduce constipation risk (21), supporting the importance of early rehabilitation. Therefore, proactive management of mobility, mood, and sleep should be considered in preventive strategies.

### 4.3 Stroke type and focus

Previous studies have shown that the incidence of constipation in patients with cerebral hemorrhage is as high as 60–80% (22). This study indicates that the incidence of new-onset constipation is elevated in patients with acute hemorrhagic stroke relative to those with ischemic stroke, supporting the findings of Wang et al. (23). This study suggests that constipation may be more prevalent in patients with cerebral hemorrhage due to the significant impairment that often limits their mobility. Furthermore, dysphagia resulting from the disease, changes in daily living activities, and the use of therapeutic medications may contribute to the onset of constipation.

This study examined the relationship between stroke focus and the occurrence of new-onset constipation following a stroke. Acute strokes in the posterior circulation were associated with a higher likelihood of poststroke constipation. Joon Han et al. (24) previously found that damage to the insula, precentral gyrus, postcentral gyrus, and lower parietal lobule is closely related to acute constipation. At the same time, another study suggested that infratentorial lesions are an independent predictor of PSC (25). Furthermore, Chen et al. (26) proposed that stroke patients with brainstem lesions are more prone to constipation. This may be attributed to impairment of the gut-brain afferent pathway, where the brainstem serves as a convergence point for rectal stimulation pathways. Damage to brainstem neurons may result in disrupted or decreased afferent signaling, elevated rectal sensation threshold, and constipation. The substantia nigra, a critical nucleus in the posterior circulation, is involved in the nigrovagal pathway, which is associated with colon tone and motility (27, 28). Damage to the nigro-vagal system may result in diminished colonic transit and constipation. Therefore, it is hypothesize that neuronal injury in the posterior circulation following an acute stroke may induce or prolong constipation by influencing the vagal route. We acknowledge that the current investigation did not perform detailed lesion mapping or subclassify posterior circulation territories (e.g., brainstem, cerebellum, thalamus). Therefore, the precise neuroanatomical mechanisms underlying the observed association remain speculative. Our findings should be interpreted as hypothesis-generating, and future studies incorporating high-resolution neuroimaging with detailed lesion localization are warranted to clarify the specific neural pathways involved.

### 4.4 Diabetes

Consistent with previous findings, these results showed that diabetes is a risk factor for refractory constipation (29). A significant number of diabetes patients experience neuropathy, which frequently results in reduced colonic transit time and the loss of the gastrocolic reflex (30). A previous research study indicates that in diabetic individuals, the tone of the anal sphincter decreases while the sensory threshold for rectal distension rises, resulting in constipation (31). As a result, diabetes may increase the risk of PSC.

### 4.5 Medication use

Our findings indicated that using osmotic diuretics may increase the risk of PSC, consistent with previous research by Su et al. (32). Osmotic diuretics promote water loss, which may lead to dehydration and increased colonic water absorption, thereby inducing or exacerbating constipation (33, 34). However, the possibility of reverse causality cannot be entirely excluded—patients with more severe stroke are more likely to receive osmotic diuretics for cerebral edema management, and such patients also have a higher baseline risk of constipation due to immobility and other factors. In our multivariable analysis, we adjusted for stroke severity (NIHSS) and functional status (Barthel Index) to mitigate this potential bias. Nevertheless, residual confounding may still exist, and the observed association should be interpreted with caution. Antacid use may elevate the risk of developing new-onset constipation after an acute stroke. Proton pump inhibitors are recognized as the most effective agents for inhibiting gastric acid secretion, with commonly used drugs such as omeprazole, rabeprazole, and pantoprazole (35). Previous clinical studies have suggested that long-term use of proton pump inhibitors can induce hypomagnesemia, which can contribute to constipation (36). A retrospective study examining the safety of long-term proton pump inhibitor use in children identified constipation and diarrhea as the most prevalent adverse events associated with the medication (37).

### 4.6 Impact of sleep disorders

This study identified that 226 out of 600 patients (37.7%) experienced daily sleep disorders. Furthermore, our multi-factor binary logistic regression findings suggested that Difficulty falling asleep was more likely to contribute to the occurrence of PSC. A recent meta-analysis indicated a correlation between sleep disorders and an increased risk of constipation. Insomnia, inadequate sleep quality, and insufficient sleep duration are associated with an increased likelihood of constipation (38). Previous studies have also reported a higher prevalence of sleep disorders or excessive daytime sleepiness in patients with Irritable Bowel Syndrome and Functional Constipation compared to healthy controls (39), further substantiating the role that sleep disorders play in gastrointestinal issues. Several studies have found that disturbed sleep quality, insufficient sleep duration, and irregular sleep schedules may interfere with circadian rhythms (40), immune function (41, 42), and hormone secretion (43), providing a mechanistic basis for the relationship between sleep problems and gastrointestinal disorders. Improving sleep habits and establishing a conducive sleep environment for stroke patients may reduce the incidence of post-stroke complications and enhance recovery outcomes.

### 4.7 Effects of depression

The current study found that mental health issues influence patients’ defecation. The multi-factor binary logistic regression analysis revealed that depressive symptoms significantly increase the risk of new-onset constipation (*p* < 0.001). This is in good agreement with the results of a previous study indicating a strong association between depression and the onset of chronic constipation (44). A large-scale cross-sectional study from the United States found that individuals with constipation are significantly more likely to suffer from major depressive disorder compared to those without constipation (45). The relationship between depression and constipation may be associated with issues with the brain-gut-microbiota axis. Jiajing Liang (46) et al. found that patients with depression had a lower relative abundance of g_Pseudoramibacter-Eubacterium and g_Candidatus-Solibacter. This indicates that gut microbiota composition may play a role in the increased prevalence of depression among patients with constipation. Subsequent studies suggest that fecal microbiota transplantation may mitigate constipation and depressive symptoms in patients by lowering serum 5-HT levels and altering the gut microbiota composition (47). Addressing mental health concerns may decrease the incidence of PSC and enhance the quality of life for patients.

### 4.8 Impact on rehabilitation

After full adjustment for confounders, new-onset constipation remained independently associated with poor discharge outcome, particularly in patients with moderate stroke severity. This confirms that the link between constipation and poor short-term prognosis is not merely a reflection of greater stroke severity or pre-existing disability. The stronger association in the moderate subgroup may represent a window of opportunity where constipation is a modifiable factor influencing recovery—in mild stroke, prognosis is excellent, while in severe stroke, outcome is largely determined by the initial brain injury. These findings highlight the need to integrate proactive bowel management into stroke rehabilitation, especially for patients with moderate deficits.

Constipation can impede stroke rehabilitation through multiple pathways. Physical discomfort and the need for assisted defecation may reduce patients’ motivation and interrupt therapy sessions. Straining during defecation increases intra-abdominal and intracranial pressure, posing risks for patients with hemorrhagic stroke or unstable cardiovascular status. A recent network meta-analysis demonstrated that effective management of PSC is associated with improved clinical outcomes, highlighting bowel function as an integral component of rehabilitation (48). Conversely, examining patients who did not develop constipation offers insights into protective factors. In our cohort, patients without new-onset constipation were characterized by better functional status, preserved mobility, and intact swallowing function (Table 2). Physical activity has been shown to reduce constipation risk (49), suggesting that early mobilization may serve as a preventive strategy. Additionally, the lower prevalence of depression and sleep disturbances in the non-constipated group highlights the role of psychological well-being, consistent with emerging evidence linking the brain-gut-microbiota axis to bowel function (50). Therefore, proactive bowel management should be integrated into stroke rehabilitation protocols (51). This bidirectional relationship between constipation and rehabilitation underscores the need for comprehensive care approaches.

### 4.9 Strengths and limitations

This study presents a systematic cross-sectional survey of constipation following acute stroke. The findings demonstrate that constipation is common in stroke patients and may occur early in the post-stroke phase. Constipation adversely affects the subsequent management and rehabilitation of stroke patients. This indicates that the early identification and intervention of PSC are essential for improving patients’ functional disabilities and enhancing their activities of daily living. It was found that elder age, hemorrhagic stroke, posterior circulation stroke, diabetes, use of osmotic diuretics and antacids, use of bedpans, Difficulty falling asleep, depression, and higher NIHSS score at admission are each risk factors for new-onset constipation. Clinical practice should prioritize not only the stabilization of patients’ vital functions but also the management of sleep conditions and psychological disorders. Optimizing diuretic and antacid use, improving the defecation environment, and implementing early mobility interventions are critical strategies for reducing post-stroke constipation risk and enhancing patient outcomes. This in turn improves patients’ quality of life post-stroke and alleviates the load on their families and society.

Despite these findings, this study has certain limitations that should be addressed. The sample size of this study was inadequate, potentially limiting its ability to identify statistical differences. This study did not include long-term follow-up, limiting its ability to assess subsequent outcomes related to constipation and long-term rehabilitation in these patients. This study did not collect detailed data on patients’ dietary patterns. Emerging evidence suggests that dietary composition influences constipation risk, with Western-style high-fat diets associated with increased risk and fiber-rich diets with reduced risk (52). Given that stroke patients often experience dietary changes due to dysphagia or modified feeding routes—and dysphagia has been independently associated with constipation (53)—the absence of nutritional assessment represents an additional limitation of our study. Future research should integrate dietary evaluations to explore these interactions. This study did not examine all factors influencing PSC. Future research should determine other factors related to constipation and stroke, including relevant laboratory blood indicators and intestinal microbiota. Although we adjusted for NIHSS and Barthel Index, residual confounding from unmeasured factors—such as dysphagia, level of consciousness, hydration status, lesion size, and rehabilitation intensity—cannot be excluded. These factors may influence both constipation risk and stroke outcomes; thus, constipation may partly reflect more severe stroke rather than being an independent predictor. It should be noted, however, that the present study initially presented only crude stratified comparisons; we have since performed multivariable adjusted analyses to address potential confounding, which strengthens the validity of our conclusions regarding the impact of constipation on short-term outcome. Finally, stroke subjects were not compared with patients with other acute vascular diseases (acute myocardial infarction) or nonvascular diseases with immobilization (fracture). Therefore, it remains uncertain whether constipation during the early post-stroke period is a complication associated with other acute vascular or nonvascular diseases. A large prospective cohort study would elucidate the intrinsic association between constipation and the rehabilitation of acute stroke, potentially assisting in the identification of effective therapeutic or preventive measures.

Additionally, this study used the Rome III criteria (published in 2006) to diagnose constipation. The Rome IV criteria, released in 2016, represent the current standard. The primary reason for this choice was that Rome IV had not been widely adopted in Chinese clinical practice at the time of study design, and our data collection tools were developed based on Rome III to ensure consistency with prior research. Notably, Rome IV requires symptoms to be present for at least 3 months with onset 6 months prior, which is more stringent than Rome III. Therefore, the incidence of new-onset constipation reported in this study may be higher than if Rome IV criteria had been applied. This limitation should be considered when interpreting our findings, and future studies are encouraged to adopt the most current diagnostic standards.

## 5 Conclusion

This study enhances the understanding of PSC and its effects on stroke outcomes. The prevalence of new-onset constipation was found to be 38.8%, exceeding that of the non-stroke population. Risk factors of new-onset constipation after stroke included elder age, hemorrhagic stroke, posterior circulation stroke, diabetes, use of osmotic diuretics and antacids, reliance on bedpans, Difficulty falling asleep, depression, and higher NIHSS score at admission. Furthermore, patients who experienced early constipation following a stroke demonstrated worse activities of daily living and greater functional impairment at discharge, suggesting that post-stroke constipation serves as a predictor of inadequate short-term rehabilitation outcomes. Monitoring patients with constipation following a stroke is crucial for effective rehabilitation management. Future research should further investigate the impact of effective post-stroke constipation management on rehabilitation outcomes.

## Data Availability

The original contributions presented in the study are included in the article/supplementary material, further inquiries can be directed to the corresponding authors.

## Footnotes

1
https://www.chictr.org.cn/showproj.html?proj=219778
